# Large-scale study in Chengdu, China: The prevalence of myopia full-correction decreased with increasing myopia in adolescents

**DOI:** 10.1016/j.heliyon.2024.e31593

**Published:** 2024-05-20

**Authors:** Jing Wei, Xiaohong Xiang, Pengbo Zhang, Jinyu Mu, Hongbin Lv, Junguo Duan

**Affiliations:** a Eye School of Chengdu University of TCM, Key Laboratory of Sichuan Province Ophthalmopathy Prevention & Cure and Visual Function Protection with TCM Laboratory, Retinal Image Technology and Chronic Vascular Disease Prevention & Control and Collaborative Innovation Center, Chengdu, China; b Ineye Hospital of Chengdu University of TCM, Sichuan Integrated Traditional Chinese and Western Medicine Myopia Prevention and Treatment Center, Sichuan Vision Protection Science Popularization Base, Key Research Laboratory of Visual Function Protection, State Administration of TCM, Chengdu, China; c Department of Ophthalmology in the Affiliated Hospital of Southwest Medical University, Luzhou, China; d Department of Ophthalmopathy, West China Hospital, Sichuan University, Chengdu, China

**Keywords:** Myopia, Spectacle wear, Myopia full-correction, Epidemiology, Children and adolescents

## Abstract

Myopia is an increasingly serious health issue among children and adolescents worldwide. This study investigated the situation related to myopia among students in Chengdu, a city in western China, and analyzed the prevalence of myopia spectacle wear and myopia full-correction and their influencing factors to understand the current status of myopia prevention. This school-based cross-sectional study investigated 1582 schools in seven districts of Chengdu City, China, enrolling a total of 417,337 students aged 6–18 years (elementary, middle, and high school) from 2020 to 2022. Examination items included uncorrected visual acuity (UCVA), slit lamp examination and non-cycloplegic autorefraction. Myopia was defined as non-cycloplegic SE ≤ −0.50 D + UCVA> 0 log MAR (age ≥6). The prevalence of myopia spectacle wear is defined as the number of people wearing glasses for myopia/the number of people with myopia (%) within the study population, and myopia full-correction is defined as normal vision after wearing glasses for myopia (≤0 log MAR for 6 years and above). With the support of the government, this programme is conducted 1–2 times a year. Statistical analyses are conducted to determine the association between myopia and various parameters.

The average age of the entire survey population was 10.96 ± 3.5 years, and the overall prevalence of myopia was 48.7%, myopia spectacle wear was 65.7%, and myopia full-correction was 50.5%. With increasing age and educational levels, the prevalence of moderate to high myopia, the prevalence of myopia spectacle wear, and the prevalence of myopia full-correction all rise. The prevalence of mild myopia full-correction (46.5%) was higher than that for moderate myopia (47.1%) and even higher than that for high myopia (39.6%). The correct utilization rate of myopic spectacles was 33.17%, increasing with age and education levels, with the highest correct utilization rate of 40.7% among those with moderate myopia. The prevalence of myopia among children and adolescents in Chengdu is relatively low, and the prevalence of myopia spectacle wear and myopia full-correction need to be improved, and it was found that with the increase of myopia, the prevalence of myopia full-correction among adolescents decreased instead.

## 1 Introduction

Myopia is a significant contributor to diminished vision and irreversible sight-threatening conditions, such as retinal detachment [1]. Myopia has emerged as a prevalent and frequently occurring condition that hampers the healthy development of teenagers. It not only significantly impacts the academic and daily life of primary and secondary school students but also poses varying degrees of obstacles to their physical and mental well-being [2,3]. Adolescent myopia is irreversible and exhibits characteristics including an earlier age of onset, heightened incidence, and rapid progression. It advances at a rate of approximately 0.75D per year [[4], [5], [6]], with high myopia progressing even more swiftly [7,8]. This condition can lead to severe complications such as retinal detachment, macular degeneration, glaucoma, and cataracts, posing a significant threat to visual health [[9], [10], [11]].

The World Vision Report published by the World Health Organization in 2020 indicates that approximately 2.6 billion people worldwide are affected by myopia [12], with 312 million individuals under the age of 19 [13]. In the United States, the prevalence of myopia among adults has increased to about 40%, up from 25% in 1971. A similar upward trend has been observed in the UK [12]. The prevalence of myopia is higher in Asia compared to Europe and the United States. For instance, in Singapore, often referred to as the "myopia capital of the world," the myopia rate among young individuals is approximately 80% [14]. According to estimates found in published literature, the global prevalence of myopia is projected to reach 50% by 2050, impacting nearly 5 billion people worldwide [15]. A recent review by Grzybowski et al. [16]. Revealed that the prevalence of myopia in school-age children is 73% in East Asia, surpassing rates observed in other regions [16]. According to epidemiological data, the overall myopia rate among Chinese children and adolescents rose from 50.2% in 2019 to 52.7% in 2020. The situation concerning myopia in this demographic remains less than optimistic. There is an overarching trend of myopia being more prevalent at a younger age and progressing at a faster rate [17]. Myopia has evolved into a significant public health concern in China and globally, posing a serious threat to the health and quality of life of affected individuals. Addressing myopia typically involves the use of corrective lenses, which can have implications for both family dynamics and socioeconomic status [18]. Examining the prevalence of myopia spectacle wear can assist governments and health insurance agencies in more effectively planning and managing medical resources, ensuring that individuals receive suitable vision correction. Analyzing the prevalence of myopia full-correction can aid educational institutions in comprehending the prevalence of vision issues among students. This understanding allows them to implement appropriate educational measures, such as enhancing vision screening and support services [19].

While the prevalence of myopia among teenagers has been documented in some major cities in China [[20], [21], [22], [23], [24]], the results of large-scale vision screening among adolescents in Chengdu are still pending. Large-scale studies on the prevalence of myopia spectacle wear and the prevalence of myopia full-correction among adolescents in western Chinese cities have not been reported to date. Chengdu, as one of the four most populous cities in China, holds significance in screening the vision of teenagers in the region. Investigating the prevalence of myopia spectacle wear and the prevalence of myopia full-correction interventions and establishing refractive files can enhance our understanding of the eye conditions among teenagers in the western part of the country. This information can further contribute to the region by providing a reference basis for formulating personalized myopia prevention and control plans for students.

## 2 Methods

### 2.1 Study population

This study, conducted from 2020 to 2022, was a school-based cross-sectional investigation aimed at comprehending the vision status of students in Chengdu, China.

Understand the prevalence of myopia and treatment gaps in urban areas in the central and western regions to provide a basis for carrying out myopia prevention and science popularization work. The method was to use primary and secondary schools aged 6–18 in 12 districts of Chengdu as survey points, and used random cluster sampling to determine which of them were Seven districts serve as the survey population, and eye examinations were conducted by uniformly trained optometrists and ophthalmologists. Each district required a sample size of more than 50,000 people. A total of 421,582 people were surveyed in the seven districts, and 2409 people had incomplete information. According to the inclusion and exclusion criteria, 1836 people were excluded and 417,337 people were included. A total of 417,337 students aged 6–18 years were included in the study, encompassing 1582 schools across seven districts in Chengdu City. The inclusion criteria comprised school students in Chengdu (including primary, junior high, and high school students) within the age range of 6–18 years. Exclusion criteria were as follows: (1) Patients with various types of glaucoma, corneal diseases, lens diseases, retinal diseases, optic nerve diseases, etc.; (2) Patients with amblyopia, strabismus, anisometropia, or severe visual impairment; (3) Individuals with trichiasis, severe conjunctivitis, etc.; (4) Those with poor compliance, mental illness, or cognitive impairment. Ethical approval for this study was granted by the Institutional Review Board of Ineye Hospital of Chengdu University of TCM, with the ethics number 2019yh-007. The study adhered to the principles outlined in the Declaration of Helsinki and was conducted with the consent of the Chengdu Municipal Education Committee. Additionally, written informed consent was obtained from at least one parent or legal guardian of each participant.

### 2.2 Equipment and research procedures

Qualified ophthalmologists and optometrists performed a comprehensive eye examination for each student, assessed their eyelids, conjunctiva, cornea, and pupil light reflection. The vision test takes place in a well-illuminated area (lux >500). Every student undergoes testing for uncorrected visual acuity (UCVA), and if they wear glasses, their vision with glasses is also assessed. Distance visual acuity was examined using the light-box E-word standard logarithmic visual acuity chart (GB 11533). The distance between the visual acuity chart and the student is 5 m, calculated using the following formula: visual acuity = distance between the subject and the visual acuity chart (m)/5 m x 0.1. A certified optometrist administered a computerized vision examination for each student, utilizing equipment such as the Topcon Auto Refractor Keratometer (KR-1, Beijing Topcon Trading Co., Ltd.), Nidek Auto Refractor Keratometer (ARK-1, Nidek Co., Ltd., Japan), and Super Vision Auto Refractor Keratometer (ARK-1, Anhui Super Vision Optical Technology Development Co., Ltd.).

Throughout the examination, each eye underwent three measurements, and the average of these measurements was recorded as the final examination result.

### 2.3 Research item definition

The occurrence of refractive errors was assessed through the spherical equivalent (SE), determined using non-cycloplegic autorefraction and uncorrected visual acuity (UCVA) values. The criteria used for defining myopia were non-cycloplegic spherical equivalent (SE) ≤ −0.50 D and uncorrected visual acuity (UCVA) > 0 log MAR (age ≥6) [25]; Hyperopia: SE ≥+ 0.50 D [26]; Astigmatism ≤ −0.75 DC [26]. The severity of myopia is categorized into low myopia (−3.00 D < SE ≤ −0.50 D), moderate myopia (−6.00 D < SE ≤ −3.00 D), and high myopia (SE ≤ −6.00 D). The prevalence of myopia spectacle wear = the number of people wearing spectacles for myopia/The number of people with myopia (%). The prevalence of myopia full-correction = normal vision after wearing spectacles for myopia (6 years and above ≤0 log MAR)/the number of people wearing spectacles for myopia (%). The correct utilization rate of myopia spectacles = normal vision after wearing spectacles for myopia (6 years and above ≤0 log MAR)/the number of people with myopia (%)

### 2.4 Data analysis

All data were analyzed statistically using SPSS 26.0 software. Count data were described using absolute numbers and constituent ratios, and chi-square tests were used to compare differences in count data. After the measurement data conforms to the normal distribution, it was expressed as the mean ± standard deviation. The measurement data was compared using the *t*-test or analysis of variance. The non-normal parameter description is expressed by the median and quartiles [*M* (*P*_*25*_*, P*_*75*_)]. Comparison use the median test. Inspection level α = 0.05. The SE correlation of the left and right eyes was r = 0.88, and the SE of both eyes was highly correlated, so this article selected the right eye for the following data analysis.

## 3 Results

### 3.1 Basic information of subjects

**Fig. 1:**
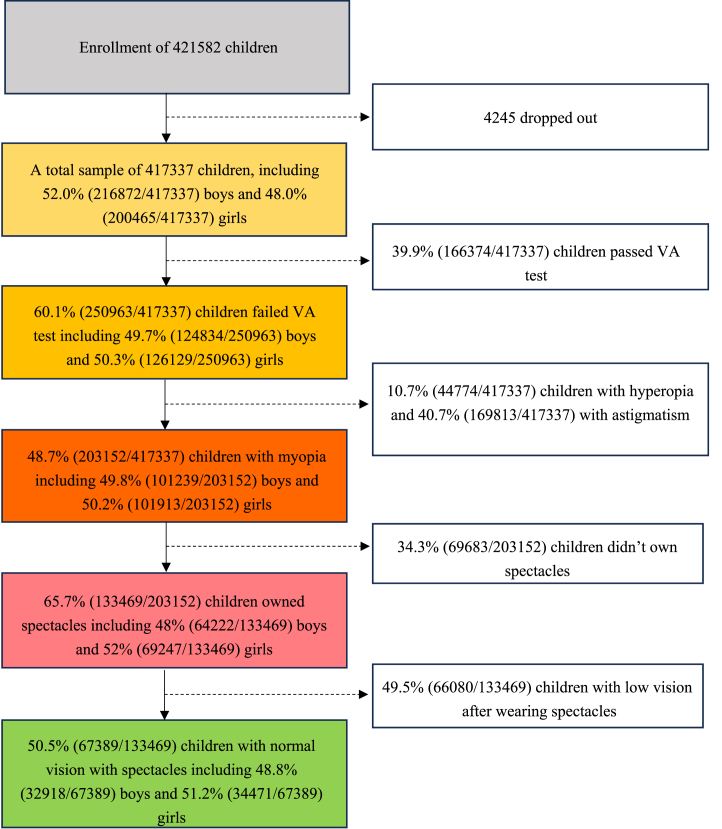
Flowchart for enrollment of children in the study.

Among the 417,337 primary and secondary school students aged 6–18 years in Chengdu, 216,872 are boys (52.0%), and 200,465 are girls (48.0%) (Mean ± standard deviation [SD]: 10.96 ± 3.5 years). 248,194 primary school students, 99,300 junior high school students, and 69,843 high school students. The distribution of individuals across all age groups and educational stages was even (Supplementary Table 1). The examination process for this study is outlined in detail in Fig. 1.

### 3.2 Subject's refractive status

**Fig. 2:**
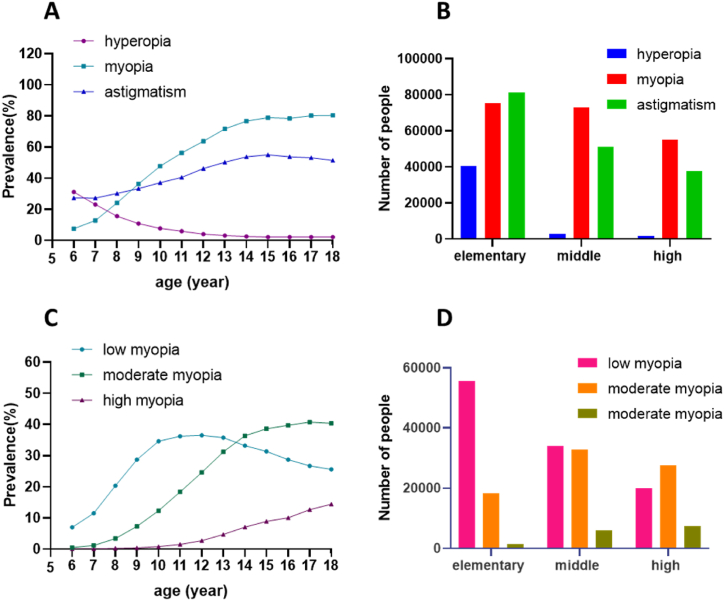
A, B Prevalence of myopia, hyperopia, and astigmatism across various age and education groups. C, D Prevalence of low, moderate, and high myopia across different age and education groups.

In this population, based on data from the right eye, the refraction examination (Supplementary Table 1 and Fig. 2A and B) revealed 203,152 individuals with myopia, resulting in a total prevalence rate of 48.7%. The myopia prevalence in boys and girls was 46.7% and 50.8%, respectively, with a statistically significant difference (P < 0.001). The prevalence of myopia increased from 7.5% to 80.4% between the ages of 6 and 18, and this age-related difference was statistically significant (P < 0.001). Individuals with varying education levels exhibited myopia prevalence rates of 30.4% in primary schools, 73.2% in junior high schools, and 78.7% in high schools, with statistically significant educational differences (P < 0.001). The examination results revealed that 44,774 individuals were hyperopia, yielding a total prevalence of 10.7%. The prevalence of hyperopia gradually decreased from 31.1% at the age of 6 to 2.1% at the age of 18, and this age-related difference was statistically significant. The prevalence of hyperopia at different educational levels was 16.3% in primary school, 2.9% in junior high school, and 2.1% in high school, with statistically significant educational differences (P < 0.001). A total of 169,813 individuals were identified with astigmatism, resulting in an overall prevalence rate of 40.7%. The prevalence of astigmatism increased from 27.4% at age 6 to a peak of 55.0% between the ages of 6 and 15, subsequently declining to 51.4% between the ages of 16 and 18. The age-related differences were statistically significant. The prevalence of astigmatism at different educational levels was 32.7% in primary school, 51.5% in junior high school, and 53.8% in high school, with statistically significant educational differences (P < 0.001).

### 3.3 Myopia degree among students aged 6–18 years old

The prevalence rates for low myopia, moderate myopia, and high myopia were 26.3%, 18.8%, and 3.6%, respectively (Table 1). There are statistically significant differences in myopia grading at different ages. The prevalence of low myopia rises with age from 6 to 12 years old, reaching a peak of 36.5% at 12 years old, and then decreases between 13 and 18 years old. In contrast, both moderate and high myopia show an increasing trend with age (Fig. 2C and D). The prevalence of myopia, low myopia, and moderate myopia exhibited statistically significant differences between genders (all P < 0.001). The prevalence of myopia was lower in boys than in girls (46.7% vs. 50.8%). Among boys, the prevalence of low myopia was lower than among girls (25.0% vs. 27.6%), and the prevalence of moderate myopia in boys was lower than in girls (18.1% vs. 19.6%). Additionally, the prevalence of high myopia was lower in boys than in girls (3.5% vs. 3.6%). There were statistically significant differences in the prevalence rates of myopia, moderate myopia, and high myopia at different educational levels (all P < 0.001) (see Table 2).

**Table 1:** Prevalence rates of low, moderate, and high myopia among primary school, junior high school, and high school students in Chengdu n (%).

Participant	Characteristics	Myopia n (%)	Low myopia n (%)	Moderate myopia n (%)	High myopia n (%)
Gender	all	203152	109636 (26.3)	78616 (18.8)	14900 (3.6)
	male	101239	54249 (25.0)	39353 (18.1)	7637 (3.5)
	Female	101913	55387 (27.6)	39263 (19.6)	7263 (3.6)
	P values		<0.001^a^	<0.001^a^	<0.001^a^
Age(year)	6	3791	3517 (7.0) _a_	233 (0.5) _a_	41 (0.1) _a_
	7	5364	4820 (11.5) _b_	484 (1.2) _b_	60 (0.1) _b_
	8	8797	7460 (20.4) _c_	1239 (3.4) _c_	98 (0.3) _c_
	9	13637	10731 (28.7) _d_	2742 (7.3) _d_	164 (0.4) _d_
	10	16519	11980 (34.6) _e_	4246 (12.3) _e_	293 (0.8) _e_
	11	18696	12060 (36.2) _f, g_	6132 (18.4) _f_	504 (1.5) _f_
	12	22253	12727 (36.5) _g_	8577 (24.6) _g_	949 (2.7) _g_
	13	23322	11631 (35.8) _f_	10154 (31.2) _h_	1537 (4.7) _h_
	14	23806	10287 (33.2) _h_	11306 (36.4) _i_	2213 (7.1) _i_
	15	24311	9686 (31.4) _i_	11897 (38.6) _j_	2728 (8.9) _j_
	16	19258	7050 (28.7) _d_	9736 (39.7) _k_	2472 (10.1) _k_
	17	16865	5607 (26.7) _j_	8585 (40.8) _l_	2673 (12.7) _l_
	18	6533	2080 (25.6) _j_	3285 (40.4) _k, l_	1168 (14.4) _m_
	P values		<0.001^a^	<0.001^a^	<0.001^a^
Education level	elementary school	248194	55662 (22.4) _a_	18300 (7.4) _a_	1510 (0.6) _a_
	middle school	99300	33989 (34.2) _b_	32790 (33.0) _b_	5954 (6.0) _b_
	high school	69843	19985 (28.6) _c_	27526 (39.4) _c_	7436 (10.6) _c_
	P values		<0.001^a^	<0.001^a^	<0.001^a^

The same letters a-l indicate that there was no statistically significant difference between the two groups.

a Indicates that there is statistical significance in the differences between different groups.

**Table 2:** Comparison of myopia spectacles wearing and full correction among primary school, junior high school, and high school students in Chengdu n (%).

Participant	Characteristics	Myopia n	Myopia spectacle wear n (%)	Myopia full-correction n (%)	Correct utilization of myopia spectacles n (%)
Gender	all	203152	133469 (65.7)	67389 (50.5)	67389 (33.2)
	male	101239	64222 (63.4)	32918 (51.3)	32918 (32.5)
	Female	101913	69247 (68.0)	34471 (49.8)	34471 (33.8)
	P values		<0.001^a^	<0.001^a^	<0.001^a^
Age(year)	6	3791	670 (17.7) _a_	281 (41.9) _a_	281 (7.4) _a_
	7	5364	1537 (28.7) _b_	706 (45.9) _a, b, c, d, e, f_	706 (13.2) _b_
	8	8797	3690 (42.0) _c_	1666 (45.2) _a, f_	1666 (18.9) _c_
	9	13637	6804 (49.9) _d_	3261 (47.9) _b, c, d, e, g_	3261 (23.9) _d_
	10	16519	9161 (55.5) _e_	4273 (46.6) _d, e, f_	4273 (25.9) _e_
	11	18696	10936 (58.5) _f_	5116 (46.8) _c, e, f_	5116 (27.4) _f_
	12	22253	14393 (64.7) _g_	7005 (48.7) _g_	7005 (31.5) _g_
	13	23322	16494 (70.7) _h_	7931 (48.1) _b, g_	7931 (34.0) _h_
	14	23806	17507 (73.5) _i_	8241 (47.1) _b, c, d, e_	8241 (34.6) _h_
	15	24311	18672 (76.8) _j_	10092 (54.1) _h_	10092 (41.5) _i_
	16	19258	15068 (78.2) _k_	8518 (56.5) _i_	8518 (44.2) _j_
	17	16865	13343 (79.1) _l_	7527 (56.4) _i_	7527 (44.6) _j_
	18	6533	5194 (79.5) _l_	2772 (53.4) _h_	2772 (42.4) _i_
	P values		<0.001^a^	<0.001^a^	<0.001^a^
Education level	elementary school	75472	38231 (50.7) _a_	17773 (46.5) _a_	17773 (23.6) _a_
	middle school	72733	52089 (71.6) _b_	24867 (47.7) _b_	24867 (34.2) _b_
	high school	54947	43149 (78.5) _c_	24749 (57.4) _c_	24749 (45.0) _c_
	P values		<0.001^a^	<0.001^a^	<0.001^a^

a Indicates that there is statistical significance in the differences between different groups. The same letters a-l indicate that there was no statistically significant difference between the two groups.

### 3.4 The situation of wearing spectacles and full-correction among myopic students aged 6 to 18 in Chengdu

**Fig. 3:**
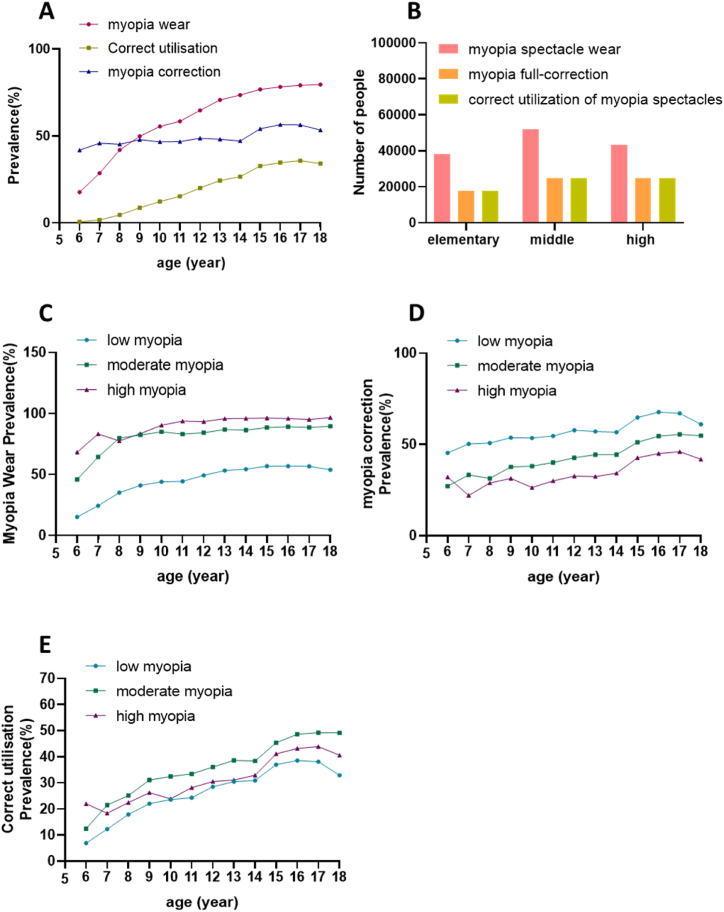
A, B The overall prevalence of myopia spectacle wear, prevalence of myopia full-correction and correct utilization rate of myopia spectacles across different age and education groups. C The prevalence of myopia spectacle wear for low, moderate, and high myopia across different age groups. D The prevalence of myopia full-correction for low, moderate, and high myopia across different age groups. E The correct utilization rate of myopia spectacles for low, moderate, and high myopia across different age groups.

Among the individuals examined, the total number with myopia was 203,152, and out of these, 133,469 wore spectacles, resulting in an overall myopia spectacle wear rate of 65.7% (Table 3). The prevalence of myopia spectacle wear among students aged 6–18 ranges from 17.67% to 79.5%. Generally, the prevalence of spectacle wear among myopic students exhibited an upward trend with age (Fig. 3A and B), and this age-related difference was statistically significant (P < 0.001). The prevalence of myopia spectacle wear for low myopia, moderate myopia, and high myopia all demonstrated an increasing trend with age, at 46.8%, 86.5%, and 95.2%, respectively (all P < 0.001) (Fig. 3C). The overall rate of wearing glasses among girls (67.95%) is higher than among boys (63.44%), with a statistically significant difference (P < 0.001). Additionally, the prevalence of myopia spectacle wear in high school (78.5%) was higher than that in junior high school (71.62%) and elementary school (50.7%), with a statistically significant difference (P < 0.001).

**Table 3:** Spectacles wearing/myopia full-correction/spectacles correcting utilization situation for low, medium and high myopia aged 6–18 years old in Chengdu n (%).

Participant	characteristics	low myopia spectacle wear n (%)	moderate myopia spectacle wear n (%)	high myopia spectacle wear n (%)	low myopia full-correction n (%)	moderate myopia full-correction n (%)	high myopia full-correction n (%)	correct utilization of low myopia spectacles n (%)	correct utilization of moderate myopia spectacles n (%)	correct utilization of high myopia spectacles n (%)
Age(year)	6	535 (15.2) _a_	107 (45.9) _a_	28 (68.3) _a_	243 (45.4) _a_	29 (27.1) _a_	9 (32.1) _a, b, c_	243 (6.9) _a_	29 (12.4) _a_	9 (22.0) a, b, c
	7	1175 (24.4) _b_	312 (64.5) _b_	50 (83.3) _a, b, c_	591 (50.3) _a, b_	104 (33.3) _a, b_	11 (22.0) _d, e_	591 (12.3) _b_	104 (21.5) _b_	11 (18.3) c
	8	2624 (35.2) _c_	990 (79.9) _c_	76 (77.6) _a, c_	1333 (50.8) _b_	311 (31.4) _a_	22 (28.9) _d, e_	1333 (17.9) _c_	311 (25.1) _b_	22 (22.4) b, c
	9	4406 (41.1) _d_	2261 (82.5) _c, d_	137 (83.5) _c_	2366 (53.7) _c_	852 (37.7) _b_	43 (31.4) _e_	2366 (22.0) _d_	852 (31.1) _c_	43 (26.2) a, b, c
	10	5285 (44.1) _e_	3611 (85.0) _e_	265 (90.4) _b, d_	2827 (53.5) _c_	1376 (38.1) _b, c_	70 (26.4) _d, e_	2827 (23.6) _e_	1376 (32.4) _c, d_	70 (23.9) c
	11	5363 (44.5) _e_	5100 (83.2) _d, f_	473 (93.8) _d, e_	2927 (54.6) _c_	2047 (40.1) _c_	142 (30.0) _d, e_	2927 (24.4) _e_	2047 (33.4) _d_	142 (28.2) b, c
	12	6274 (49.3) _f_	7232 (84.3) _e, f_	887 (93.5) _d_	3625 (57.8) _d_	3091 (42.7) _d_	289 (32.6) _d, e_	3625 (28.5) _f_	3091 (36.0) _e_	289 (30.5) a, b
	13	6195 (53.3) _g_	8827 (86.9) _g_	1472 (95.8) _e, f_	3536 (57.1) _d_	3918 (44.4) _e_	477 (32.4) _d, e_	3536 (30.4) _g_	3918 (38.6) _f_	477 (31.0) a, b
	14	5603 (54.5) _g_	9778 (86.5) _g_	2126 (96.1) _f, g_	3176 (56.7) _d_	4338 (44.4) _e_	727 (34.2) _d_	3176 (30.9) _g,h_	4338 (38.4) _f_	727 (32.9) a
	15	5517 (57.0) _h_	1052 (88.5) _h_	2626 (96.3) _f, g_	3577 (64.8) _e_	5394 (51.2) _f_	1121 (42.7) _c_	3577 (36.9) _i_	5394 (45.3) _g_	1121 (41.1) d
	16	4012 (56.9) _h_	8684 (89.2) _h_	2372 (96.0) _f, g_	2717 (67.7) _f_	4734 (54.5) _g_	1067 (45.0) _a, b_	2717 (38.5) _j_	4734 (48.6) _h_	1067 (43.2) d, e
	17	3183 (56.8) _h_	7614 (88.7) _h_	2546 (95.2) _e, g_	2133 (67.0) _f_	4222 (55.5) _g_	1172 (46.0) _b_	2133 (38.0) _i, j_	4222 (49.2) _h_	1172 (43.8) e
	18	1121 (53.9) _g_	2943 (89.6) _h_	1130 (96.7) _f_	684 (61.0) _g_	1614 (54.8) _g_	474 (41.9) _a, c_	684 (32.9) _h_	1614 (49.1) _h_	474 (40.6) d, e
	P values	<0.001^a^	<0.001^a^	<0.001^a^	<0.001^a^	<0.001^a^	<0.001^a^	<0.001^a^	<0.001^a^	<0.001^a^
	all	51293 (46.8)	67389 (86.5)	14188 (95.2)	29735 (58.0)	32030 (47.1)	5624 (39.6)	29735 (27.1)	32030 (40.7)	5624 (37.7)

a Indicates that there is statistical significance in the differences between different groups. The same letters a-l indicate that there was no statistically significant difference between the two groups.

Out of the 133,469 students wearing spectacles for myopia, 67,389 required myopia correction, resulting in a myopia full-correction rate of 50.5%. The prevalence of myopia full-correction among students aged 6–18 varied from 41.9% to 56.53% across different age groups. Notably, the prevalence of myopia full-correction generally increased with age (Fig. 3D), and this age-related difference was statistically significant (P < 0.001). The prevalence of myopia full-correction for students with various education levels were 46.5% in primary school, 47.7% in junior high school, and 57.4% in high school. This variation was statistically significant (P < 0.001). Furthermore, the prevalence of myopia full-correction differed between genders, with a rate of 49.78% for girls and 51.26% for boys, and this gender-related difference was statistically significant (P < 0.001).

Within the entire study population, the correct utilization rate of myopia spectacles stood at 33.17%. Across different age groups among students, the correct utilization rate of myopia spectacles ranged from 7.4% to 44.6% (Fig. 3E). Notably, this rate increased with age between 6 and 17 years and showed a decline at 18 years of age. The correct utilization rate of myopia spectacles varied among students with different education levels, with rates of 23.6% in primary school, 34.2% in junior high school, and 45.0% in high school. This discrepancy was statistically significant (P < 0.001). The correct utilization rate of myopia spectacles also differed based on the degree of myopia, with rates of 40.7% for moderate myopia and 27.1% for low myopia, and this difference was statistically significant (P < 0.001). Additionally, the correct utilization rate of myopia spectacles among students of different genders was 32.5% for boys and 33.8% for girls, with a statistically significant difference (P < 0.001).

In the statistical analysis of adolescents with low, moderate, and high myopia, it was observed that the prevalence of myopia spectacle wear, the prevalence of myopia full-correction, the correct utilization rate of myopia spectacles (Table 3) at different degrees of myopia generally increased with age between 6 and 17 years. In this analysis, it was observed that the prevalence of high myopia spectacle wear decreased among those aged 7–8. Regarding the prevalence of high myopia full-correction, a decrease was found between the ages of 6–7 and 9–10 years old. Additionally, the correct utilization rate of high myopia spectacles was found to be reduced between 6-7 and 9–10 years old.

### 3.5 Biological parameters of eyeballs in students aged 6–18 Years and comparison with those wearing myopia spectacles

**Fig. 4:**
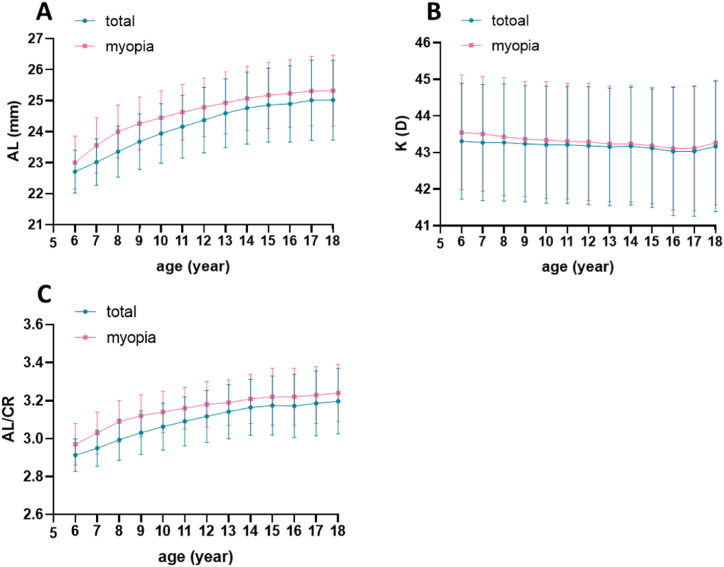
The correlation between corneal AL(A), K (B), AL/CR (C), and age.

The findings from this study reveal that, among adolescents aged 6–18, the AL and AL/CR of the eye increase with age, and this increase is more pronounced in individuals with myopia (Table 4) (Fig. 4A and C), this difference was statistically significant (P < 0.001). The corneal curvature increases with age (Fig. 4B). In myopic patients, the corneal curvature decreased from 43.55 ± 1.57 at age 6 to 43.27 ± 1.70 at age 18, and this difference was statistically significant (P < 0.001). The stratified analysis revealed that with the increase in AL, AL/CR ratio, and K, the prevalence of myopia spectacle wear and the correct utilization rate of myopia spectacles increased, while the prevalence of myopia full-correction decreased (Supplementary Table 2). In each stratified analysis, it was observed that as the eye axis, myopia axial ratio, and corneal curvature increase, the prevalence of myopia spectacle wear and the correct utilization rate of myopia spectacles were higher in girls than in boys. There was a correlation between SE and AL/CR, with a correlation coefficient (R = −0.797).

**Table 4:** Ocular biometric parameters in students aged 6–18 years (x ± s).

Participant	Characteristics	AL (mm)	K (D)	AL/CR
Gender	all	23.99 ± 1.27	43.20 ± 1.62	3.07 ± 0.16
	male	24.23 ± 1.25	42.86 ± 1.59	3.08 ± 0.16
	Female	23.73 ± 1.22	43.57 ± 1.58	3.06 ± 0.16
	P values	<0.001***	<0.001***	<0.001***
Age(year)	6	22.71 ± 0.69	43.31 ± 1.58	2.913 ± 0.09
	7	23.02 ± 0.75	43.27 ± 1.59	2.950 ± 0.10
	8	23.36 ± 0.83	43.27 ± 1.60	2.993 ± 0.11
	9	23.68 ± 0.90	43.24 ± 1.59	3.031 ± 0.12
	10	23.94 ± 0.96	43.22 ± 1.60	3.063 ± 0.12
	11	24.16 ± 1.01	43.21 ± 1.60	3.091 ± 0.13
	12	24.37 ± 1.06	43.17 ± 1.61	3.117 ± 0.14
	13	24.59 ± 1.11	43.16 ± 1.61	3.142 ± 0.14
	14	24.76 ± 1.16	43.17 ± 1.61	3.165 ± 0.15
	15	24.86 ± 1.20	43.12 ± 1.61	3.174 ± 0.15
	16	24.90 ± 1.23	43.03 ± 1.62	3.172 ± 0.17
	17	25.01 ± 1.29	43.03 ± 1.75	3.186 ± 0.17
	18	25.02 ± 1.29	43.17 ± 1.78	3.197 ± 0.17
	P values	<0.001***	<0.001***	<0.001***
Education level	elementary school	23.52 ± 1.02	1.6 ± 0.003	3.01 ± 0.13
	middle school	24.65 ± 1.14	1.6 ± 0.005	3.15 ± 0.15
	high school	24.92 ± 1.24	1.74 ± 0.007	3.18 ± 0.17
	P values	<0.001***	<0.001***	<0.001***

AL, Axial length; K, Corneal curvature; CR, corneal radius of curvature; ***Indicates that there is statistical significance in the differences between different groups.

## 4 Discussion

Among the 417,337 primary and secondary school students aged 6–18 in Chengdu, the overall prevalence of myopia was 48.7% (203,152/417,337), hyperopia was 10.7% (44,774/417,337), and astigmatism was 40.7% (169,813/417,337). As age increases, the prevalence of moderate and high myopia showed an upward trend. Among the total number of individuals with myopia, the overall prevalence of myopia spectacle wear was 65.7% (133,469/203,152), the prevalence of myopia full-correction was 50.5% (67,389/133,469), and the correct utilization rate of myopia spectacles was 33.17% (67,389/203,152). Within the overall study population, the myopia prevalence was lower compared to children in East Asia (73%), higher than that of children in Africa (10%) and North and South America (42%) [16]. It was also notably lower than the average myopia rate among Chinese children and adolescents in 2020 (52.7%), as well as lower than specific regions such as Shenzhen, a coastal city in southern China (49.4%) [27], and Yunnan, a border city in western China (48.05%) [28]. In addition to racial and genetic factors, variations in computerization and lifestyle changes may play a role in the elevated prevalence of myopia in children and adolescents compared to other regions worldwide and within other cities in the country. In the findings of this study, the prevalence of hyperopia exceeded the results of the WHO national regional statistical survey conducted by Hassan et al. (which reported an average total hyperopia rate in children of 4.6%) [29]. This suggests that the reserve level of myopia in Chengdu is considerably higher than the global average.

Our study results revealed a progressive increase in the prevalence of myopia in Chengdu with age, aligning with findings from other researches [30]. The prevalence rose from 7.5% at 6 years old to 80.4% at 18 years old. The overall myopia prevalence rate (48.7%) is lower than the survey results for the same age group of teenagers in Weifang, China (75.35%) [31]. Among them, the prevalence of myopia was observed to increase most rapidly among students aged 7–14 years old. The prevalence of moderate and high myopia showed an upward trend with age. The prevalence of low myopia gradually increased from the age of 6, peaking at the age of 12 (junior high school stage). Analyzing the reasons for these results, it was plausible that individuals with initially low myopia could transition to moderate to high myopia due to heightened academic pressure. The prevalence of high myopia (3.6%) was higher than the results reported in Xian et al.'s study in Shanghai (2.1%) but lower than the prevalence of high myopia in urban schools in Wenzhou (5.05%). One possible reason is that Xian et al.'s study focused on adolescents aged 6–14 years, whereas our research included individuals aged 6–18 years. Existing research indicates that the incidence of high myopia tends to increase with age. Hence, the observed higher incidence of high myopia in this study compared to that in Shanghai could be attributed to the age distribution of the surveyed population. Consequently, there is a need to intensify myopia prevention and control measures among adolescents of this age group, aiming to mitigate the overall myopia prevalence and reduce the occurrence of moderate to high myopia.

The overall myopia rate and the prevalence rates of low, moderate, and high myopia were lower in boys compared to girls, aligning with prior research findings and suggesting that girls face a higher risk of developing myopia [32,33]. Due to the influence of growth hormone and estrogen, girls tend to mature earlier than boys and enter puberty at an earlier age [34]. This phenomenon may contribute to the higher prevalence of myopia in girls compared to boys at a certain age. Moreover, despite the government's repeated calls for schools and parents to alleviate the academic burden on students, Chinese students still face substantial academic and educational pressures. Additionally, girls tend to dedicate more time to meticulous reading and writing, with less time allocated to outdoor activities, while boys, in general, exhibit more active lifestyles.

This study revealed an overall prevalence of myopia spectacle wear of 65.7% among individuals with myopia. The prevalence of myopia spectacle wear gradually increased with age, educational level, and degree of myopia, ranging from 17.7% at 6 years old to 79.5% at 18 years old. In terms of educational progression, the rate climbed from 50.7% in primary school to 78.5% in high school. Notably, the rate elevated from 46.8% for low myopia to 95.2% for high myopia. This was considerably higher than the prevalence of myopia spectacle wear reported in rural areas of Yunnan, China (35.6%) [18], and among migrant children in cities in eastern China (35.3%) [35]. The prevalence of myopia full-correction exhibited a gradual increase with age and education level, with the most significant growth observed between the ages of 14 and 15, followed by a decline at the age of 18. The decreased at age 18 could be attributed to considerations related to future career planning, as some senior high school students opt for corneal refractive surgery to address myopia. Interestingly, there is a notable decrease in the rate of myopia correction as the degree of myopia increases, with rates of 58.0% for low myopia, 47.1% for moderate myopia, and 39.7% for high myopia. This trend might be attributed to a prevailing misconception among parents that undercorrection is beneficial in slowing down the progression of myopia. Gender disparities were observed in the impact on the prevalence of myopia spectacle wear and myopia full-correction. While the rate of boys wearing spectacles was lower than that of girls, the prevalence of myopia full-correction for boys was significantly higher than that for girls. This suggested that boys had a greater demand for clear vision through glasses. Additionally, the correct utilization rate of myopia spectacles in junior high schools and high schools (34.2%, 45.0%) was lower than the correction rate reported in Wenzhou (39.22%, 53.49%) [36].

Among various degrees of myopia, the highest correct utilization rate of myopia spectacles was observed for moderate myopia at 40.7%. Analyzing the reasons, for low-degree myopia, patients have a lower degree and, consequently, a reduced need for optimal visual correction. Conversely, in high-degree myopia, patients with excessively high myopia may experience lower letter contrast sensitivity and diminished subjective visual function [3]. At the same time, the number of individuals with moderate myopia increases most rapidly at the age of 12–13, which corresponds to the second stage of puberty. During this period, the shape and function of the body undergo further development, including advancements in eye-brain coordination and visual function. Therefore, individuals with moderate myopia exhibit a higher demand for visual quality, resulting in the highest correct utilization rate of myopia spectacles. Although, considering the overall trend, the prevalence of myopia spectacle wear, the prevalence of myopia full-correction and the correct utilization rate of myopia spectacles for low, moderate, and high myopia generally increased with age between 6 and 17 years. It was observed that the prevalence of high myopia spectacle wear decreased at ages 7–8, the prevalence of high myopia full-correction decreased at ages 6–7 and 9–10, and the correct utilization rate of high myopia spectacles decreased at ages 6–7 and 9–10. These findings highlight the importance of focusing on eyeglass-wearing interventions, particularly for highly myopic children aged 6–10, in children's myopia management programs. It underscores the need for the development of appropriate eyeglass-wearing strategies.

As age increases, the AL and AL/CR gradually increase. Among the total study population, the increase in AL and AL/CR was more obvious in patients with myopia. Research indicates a close association between AL, AL/CR, and the progression of myopia, affirming that ''as age increases, the prevalence of myopia also increases.'' Consequently, our study results were in line with findings from prior research. In this study, we conducted a comprehensive analysis of the impact of changes in AL, the AL/CR ratio, and K on the glasses-wearing rate. Given their association with the spherical equivalent (SE), our findings revealed that as AL, the AL/CR ratio, and K increased, the glasses-wearing rate and the rate of glasses-wearing for full correction among teenagers in Chengdu also increased. This suggestes a conscious effort among parents in the region to manage and control the progression of refractive and axial myopia.

The strengths of this study were noteworthy. Firstly, the substantial sample size encompassed all children and adolescents aged 6–18 years in Chengdu. Notably, there is a scarcity of domestic reports on the refractive status, the prevalence of myopia spectacle wear, and the prevalence of myopia full-correction among adolescents in southwest China. This study markes the first comprehensive documentation of the widespread use and correct utilization of myopia spectacles among Chinese adolescents.

Due to time and location constraints, cycloplegic autorefraction was not used. Studies have shown that non-cycloplegic autorefraction finds a prevalence of myopia twice as high as than found with cycloplegia. Therefore, it can be speculated that after cycloplegia, the prevalence of myopia in Chengdu adolescents and children will be lower. Secondly, this study was a cross-sectional study and cannot assess the causal relationship between risk factors, refractive status, and glasses-wearing rate in children and adolescents. We will strengthen the longitudinal cohort study in later research to more accurately and scientifically analyze data related to the refractive status, glasses-wearing rate and full correction rate of different ethnic groups, so as to provide targeted scientific basis for preventing and controlling the occurrence and development of myopia.

To sum up, the prevalence of myopia among children and adolescents aged 6–18 years in Chengdu was relatively low (48.7%). compared with the national prevalence of myopia among children and adolescents released by China in 2020 (52.7%) [16], the rate of wearing glasses was high (65.7%) and the rate of full correction was high (50.5%). Age, gender, and education level were all influencing factors of myopia. Age, gender, degree of myopia, AL and AL/CR were important factors affecting the rate of myopia wearing glasses and full correction rate. These results showed that Chengdu had a strong awareness of adolescent eye health management and adequate science popularization work. However, it is still necessary to continue to maintain and strengthen the promotion of myopia work in adolescents and children. It is important to take a series of measures to plan comprehensive eye care services, including popular science education, campus lectures, improving community vision examinations and refractive services, such as glasses, and managing and preventing myopia-related ocular complications and vision loss in patients with high myopia. Not only that, in the daily life of every family, the knowledge about myopia and glasses should be popularized among teenagers, children and parents.

Hence, this study revealed that the overall myopia prevalence among adolescents aged 6–18 in Chengdu, China, stands at 48.7%. The prevalence of myopia spectacle wear showed a gradual increase with age, yet ultimately, 20.5% still abstain from wearing spectacles. Additionally, the prevalence of myopia full-correction was 53.37%. There is considerable ground to cover in addressing myopia correction, and the spectacle market awaits regulatory measures.

Solving the issue of using spectacles for myopia and implementing standardized myopia correction are challenging tasks, and further strides must be taken to ensure the effective management of myopia.

## Institutional Review Board statement

The study was conducted according to the guidelines of the Declaration of Helsinki. Ethical approval for this study was granted by the Institutional Review Board of Ineye Hospital of 10.13039/501100008402Chengdu University of TCM, with the ethics number 2019yh-007.

## Informed consent statement

Informed consent was obtained from all participants involved in the study.

## Data availability statement

The data presented in this study are available upon request from the corresponding authors.

## CRediT authorship contribution statement

**Jing Wei:** Writing – review & editing, Writing – original draft, Visualization, Supervision, Project administration, Formal analysis, Conceptualization. **Xiaohong Xiang:** Supervision. **Pengbo Zhang:** Methodology. **Jinyu Mu:** Project administration, Investigation. **Hongbin Lv:** Visualization, Supervision, Software. **Junguo Duan:** Data provision, Data curation, Investigation, Resources, Funding acquisition.

## Declaration of competing interest

The authors declare that they have no known competing financial interests or personal relationships that could have appeared to influence the work reported in this paper.
