# Supplementary material for: Large-scale study in Chengdu, China: The prevalence of myopia full-correction decreased with increasing myopia in adolescents
Source: Heliyon. 2024 May 20;10(11):e31593. doi: 10.1016/j.heliyon.2024.e31593 (PMC11152689; doi:10.1016/j.heliyon.2024.e31593)
Supplement: Multimedia component 1 [file mmc1.docx]

| Supplementary table 1: Refractive status of primary school, junior high school, and high school students in Chengdu n (%). | | | | |  |
| --- | --- | --- | --- | --- | --- |
| Participant | Characteristics | N | Hyperopia n (%) | Myopia n (%) | Astigmatism n (%) |
|  | all | 417337 | 44774 (10.7) | 203152 (48.7) | 169813 (40.7) |
| Gender | male | 216872 | 23914 (11.0) | 101239 (46.7) | 90518 (41.7) |
|  | Female | 200465 | 20860 (10.4) | 101913 (50.8) | 79295 (39.6) |
|  | P values |  | <0.001*** | <0.001*** | <0.001*** |
| Age(year) | 6 | 50454 | 15706 (31.1) _a_ | 3791 (7.5) _a_ | 13822 (27.4) _a_ |
|  | 7 | 41991 | 9716 (23.1) _b_ | 53641 (2.8) _b_ | 11422 (27.2) _a_ |
|  | 8 | 36556 | 5696 (15.6) _c_ | 8797 (24.1) _c_ | 11051 (30.2) _b_ |
|  | 9 | 37427 | 4039 (10.8) _d_ | 13637 (36.4) _d_ | 12509 (33.4) _c_ |
|  | 10 | 34651 | 2678 (7.7) _e_ | 16519 (47.7) _e_ | 12850 (37.1) _d_ |
|  | 11 | 33296 | 1959 (5.9) _f_ | 18696 (56.2) _f_ | 13497 (40.5) _e_ |
|  | 12 | 34888 | 1423 (4.1) _g_ | 22253 (63.8) _g_ | 16112 (46.2) _f_ |
|  | 13 | 32531 | 1022 (3.1) _h_ | 23322 (71.7) _h_ | 16348 (50.3) _g_ |
|  | 14 | 31031 | 773 (2.5) _i_ | 23806 (76.7) _i_ | 16701 (53.8) _h_ |
|  | 15 | 30801 | 633 (2.1) _j_ | 24311 (78.9) _j_ | 16947 (55.0) _i_ |
|  | 16 | 24552 | 518 (2.1) _j_ | 19258 (78.4) _j_ | 13214 (53.8) _h_ |
|  | 17 | 21037 | 439 (2.1) _j_ | 16865 (80.2) _k_ | 11163 (53.1) _h_ |
|  | 18 | 8122 | 172 (2.1) _i, j_ | 6533 (80.4) _k_ | 4177 (51.4) _g_ |
|  | P values |  | <0.001*** | <0.001*** | <0.001*** |
| Education level | elementary school | 248194 | 40424 (16.3) _a_ | 75472 (30.4) _a_ | 81111 (32.7) _a_ |
|  | middle school | 99300 | 2871 (2.9) _b_ | 72733 (73.2) _b_ | 51092 (51.5) _b_ |
|  | high school | 69843 | 1479 (2.1) _c_ | 54947 (78.7) _c_ | 37610 (53.8) _c_ |
|  | P values |  | <0.001*** | <0.001*** | <0.001*** |

***Indicates that there is statistical significance in the differences between different groups. The same letters a-l indicate that there was no statistically significant difference between the two groups.

| Supplementary table 2: Stratification of ocular biometric parameters and myopia spectacle wear/myopia full-correction/correct utilization of myopia spectacles among students aged 6-18 years in Chengdu n (%). | | | | | | | | | | |
| --- | --- | --- | --- | --- | --- | --- | --- | --- | --- | --- |
| Participant | characteristics | myopia spectacle wear n (%) | myopia spectacle wear n (%)-Male | myopia spectacle wear n (%)-Famale | myopia full-correction n (%) | myopia full-correction n (%) -Male | myopia full-correction n (%) -Famale | correct utilization of myopia spectacles n (%) | correct utilization of myopia spectacles n (%) -Male | correct utilization of myopia spectacles n (%) -Famale |
| AL (mm) | ≤22 | 881 (14.6) _a_ | 162 (14.8) _a_ | 719 (14.6) _a_ | 129 (49.6) _a, b, c_ | 24 (58.3) _a, b, c_ | 105 (47.6) _a, b, c_ | 881 (7.3) _a_ | 162 (8.6) _a_ | 719 (7.0) _a_ |
|  | 22＜ ≤23 | 7898 (22.0) _b_ | 2082 (15.2) _b_ | 5816 (24.4) _b_ | 1734 (58.7) _e_ | 316 (58.2) _c, d_ | 1418 (58.7) _e_ | 7898 (12.9) _b_ | 2082 (8.8) _a_ | 5816 (14.3) _b_ |
|  | 23＜ ≤24 | 37907 (43.3) _c_ | 12269 (31.4) _c_ | 25638 (49.1) _c_ | 16423 (56.8) _d, e_ | 3847 (59.9) _d_ | 12576 (55.9) _d_ | 37907 (24.6) _c_ | 12269 (18.8) _b_ | 25638 (27.4) _c_ |
|  | 24＜ ≤25 | 73129 (63.2) _d_ | 33720 (52.8) _d_ | 39409 (72.1) _d_ | 46202 (52.6) _c_ | 17800 (55.7) _c_ | 28402 (50.7) _c_ | 73129 (33.3) _d_ | 33720 (29.4) _c_ | 39409 (36.6) _d_ |
|  | 25＜ ≤26 | 55614 (79.2) _e_ | 33158 (74.2) _e_ | 22456 (86.5) _e_ | 44022 (49.1) _b_ | 24593 (51.1) _b_ | 19429 (46.5) _b_ | 55614 (38.8) _e_ | 33158 (37.9) _d_ | 22456 (40.2) _e_ |
|  | ＞26 | 27723 (90.0) _f_ | 19848 (88.9) _f_ | 7875 (92.9) _f_ | 24959 (44.3) _a_ | 17642 (45.0) _a_ | 7317 (42.7) _a_ | 27723 (39.9) _f_ | 19848 (40.0) _e_ | 7875 (39.6) _e_ |
|  | P values | <0.001*** | <0.001*** | <0.001*** | <0.001*** | <0.001*** | <0.001*** | <0.001*** | <0.001*** | <0.001*** |
| K (D) | ≤41 | 15402 (61.0) _a_ | 10596 (60.9) _a_ | 4806 (61.2) _a_ | 9393 (52.3) _a_ | 6453 (52.2) _a_ | 2940 (52.6) _a_ | 15402 (31.9) _a_ | 10596 (31.8) _a_ | 4806 (32.1) _a, b_ |
|  | 41＜ ≤42 | 27232 (64.7) _b_ | 17313 (63.4) _b, c_ | 9919 (67.1) _b_ | 17628 (52.1) _a_ | 10975 (52.2) _a_ | 6653 (51.9) _a_ | 27232 (33.7) _b, c_ | 17313 (33.1) _b_ | 9919 (34.8) _c, d_ |
|  | 42＜ ≤43 | 47077 (65.9) _c_ | 26128 (63.8) _b_ | 20949 (68.5) _c_ | 31020 (51.4) _a, b_ | 16670 (51.5) _a, b_ | 14350 (51.2) _a_ | 47077 (33.8) _c_ | 26128 (32.8) _a, b_ | 20949 (35.1) _d_ |
|  | 43＜ ≤44 | 50709 (66.4) _c, d_ | 24126 (64.0) _b, c_ | 26583 (68.5) _c_ | 33654 (50.6) _b_ | 15442 (51.7) _a, b_ | 18212 (49.7) _b_ | 50709 (33.6) _b, c_ | 24126 (33.1) _b_ | 26583 (34.0) _c, e_ |
|  | 44＜ ≤45 | 36245 (66.8) _d_ | 14382 (64.1) _b_ | 21863 (68.6) _c_ | 24210 (49.5) _c_ | 9213 (50.5) _b_ | 14997 (48.9) _b_ | 36245 (33.1) _b_ | 14382 (32.3) _a, b_ | 21863 (33.5) _b, e_ |
|  | ＞45 | 26487 (66.3) _c, d_ | 8694 (62.9) _b_ | 17793 (68.0) _b, c_ | 17564 (47.5) _d_ | 5469 (47.6) _c_ | 12095 (47.5) _c_ | 26487 (31.5) _a_ | 8694 (29.9) _c_ | 17793 (32.3) _a_ |
|  | P values | <0.001*** | <0.001*** | <0.001*** | <0.001*** | <0.001*** | <0.001*** | <0.001*** | <0.001*** | <0.001*** |
| AL/CR | ≤2.8 | 839 (21.2) _a_ | 334 (19.2) _a_ | 505 (22.6) _a_ | 178 (52.2) _a, b, c_ | 64 (53.1) _a, b, c_ | 114 (51.8) _a, b, c_ | 839 (11.1) _a_ | 334 (10.2) _a_ | 505 (11.7) _a_ |
|  | 2.8＜ ≤3.0 | 16250 (26.6) _b_ | 6868 (24.2) _b_ | 9382 (28.4) _b_ | 4329 (59.4) _c_ | 1663 (60.4) _d_ | 2666 (58.7) _c_ | 16250 (15.8) _b_ | 6868 (14.6) _b_ | 9382 (16.7) _b_ |
|  | 3.0＜ ≤3.2 | 106953 (56.9) _c_ | 50711 (51.7) _c_ | 56242 (61.7) _c_ | 60899 (55.2) _b_ | 26207 (56.8) _c_ | 34692 (53.9) _b_ | 106953 (31.4) _c_ | 50711 (29.3) _c_ | 56242 (33.3) _c_ |
|  | 3.2＜ ≤3.4 | 67448 (84.8) _d_ | 36291 (82.0) _d_ | 31157 (88.0) _d_ | 57188 (46.7) _a_ | 29769 (48.1) _b_ | 27419 (45.2) _a_ | 67448 (39.6) _d_ | 36291 (39.5) _d_ | 31157 (39.8) _d_ |
|  | 3.4＜ ≤3.5 | 8256 (92.8) _e_ | 4935 (92.0) _e_ | 3321 (94.0) _e_ | 7663 (41.3) _d_ | 4541 (42.3) _a_ | 3122 (39.7) _d_ | 8256 (38.3) _e_ | 4935 (38.9) _d_ | 3321 (37.3) _e_ |
|  | ＞3.5 | 3406 (94.3) _f_ | 2100 (94.2) _f_ | 1306 (94.5) _e_ | 3212 (39.5) _d_ | 1978 (38.7) _e_ | 1234 (40.8) _d_ | 3406 (37.2) _e_ | 2100 (36.4) _e_ | 1306 (38.5) _d, e_ |
|  | P values | <0.001*** | <0.001*** | <0.001*** | <0.001*** | <0.001*** | <0.001*** | <0.001*** | <0.001*** | <0.001*** |

AL, Axial length; K, Corneal curvature; CR, corneal radius of curvature

***Indicates that there is statistical significance in the differences between different groups. The same letters a-l indicate that there was no statistically significant difference between the two groups.
